# 806. Burn Inducing Contact System: A Reliable Research Tool

**DOI:** 10.1093/jbcr/irag033.184

**Published:** 2026-04-06

**Authors:** Emma Blomberg, Sanaz Saleh, Michael Sneller, Amani A Bashir, Mohamad M Mokadem, Shady Al Hayek

**Affiliations:** University of Iowa Carver College of Medicine, Coralville, IA; University of Iowa, Internal Medicine Department, Iowa City, IA; Protostudios, University of Iowa, Iowa City, IA; University of Iowa Health Care, Iowa City, IA; University of Iowa Carver College of Medicine, Coralville, IA; University of Iowa Health Care, Iowa City, IA

## Abstract

**Introduction:**

Burn injuries are among the leading causes of non-fatal trauma worldwide and are marked by a complex systemic response that affects other organs beyond the skin. Effective management of burn injuries relies heavily on a deep understanding of post-burn physiology, often achieved through replication of burns on animal models. Multiple rodent models have been created to replicate burn pathology in research, but a lack of standardization in injury parameters, particularly constant temperature, exposure time, and pressure, has constrained the reliability and translational relevance of this work. To address this, we present a novel burn induction device designed to create precise and consistent thermal contact in mouse models.

**Methods:**

The Burn Inducting Contact System (BICS) system was developed to induce a range of burn severities from superficial partial-thickness to full-thickness burn on the dorsum of adult wild-type mice. The device uses an Arduino to control a heating element with temperature feedback from a thermocouple, allowing the user to set and monitor heat via buttons and a screen. It utilizes a 18 V power supply to increase tool temperature and a machined metal tip to deliver consistent 2 × 2 cm burns. Burn injuries were generated through the determined combinations of temperature and time exposure: 50°C for 2 and 3 seconds, and 60°C for 2 and 3 seconds. The device was applied to the skin vertically without any additional pressure, beyond the weight of the tool. 5 hours post-burn, 10 skin biopsies were harvested from burned and non-burned surface areas for relative analysis. The tissue was then processed with H&E staining for histological analysis to confirm burn depth based on the percentage of necrotic adnexal structures and dermal involvement.

**Results:**

In our experiment, 60°C for 2 seconds provided a mixed superficial and deep partial-thickness burn. 60°C for 3 seconds induced a consistent full-thickness burn. 50°C for 2-3 seconds exposure led to superficial injuries. The device allowed for uniform and reliable thermal damage as desired. A strong association between determined parameters and histological depth demonstrates its applicability for developing burn models.

**Conclusions:**

The BICS system provides a consistent and adaptable method for inducing contact burns under controlled conditions by adjusting heat and contact duration. It represents a valuable tool for investigating burn pathophysiology and improving experimental models in translational burn research.

**Applicability of Research to Practice:**

The BICS system determines the temperature settings required to deliver consistent burn wound depths. This provides a standardized research methodology for burn studies using mouse models, ultimately enhancing the reliability of burn research and deepening the understanding of burn pathophysiology to improve the care and treatment of burn injuries.

**Funding for the study:**

N/A.

